# Flow Cytometry Bioinformatics

**DOI:** 10.1371/journal.pcbi.1003365

**Published:** 2013-12-05

**Authors:** Kieran O'Neill, Nima Aghaeepour, Josef Špidlen, Ryan Brinkman

**Affiliations:** 1 Terry Fox Laboratory, BC Cancer Agency, Vancouver, British Columbia, Canada; 2 Bioinformatics Graduate Program, University of British Columbia, Vancouver, British Columbia, Canada; 3 Department of Medical Genetics, University of British Columbia, Vancouver, British Columbia, Canada; University of Toronto, Canada

## Abstract

Flow cytometry bioinformatics is the application of bioinformatics to flow cytometry data, which involves storing, retrieving, organizing, and analyzing flow cytometry data using extensive computational resources and tools. Flow cytometry bioinformatics requires extensive use of and contributes to the development of techniques from computational statistics and machine learning. Flow cytometry and related methods allow the quantification of multiple independent biomarkers on large numbers of single cells. The rapid growth in the multidimensionality and throughput of flow cytometry data, particularly in the 2000s, has led to the creation of a variety of computational analysis methods, data standards, and public databases for the sharing of results. Computational methods exist to assist in the preprocessing of flow cytometry data, identifying cell populations within it, matching those cell populations across samples, and performing diagnosis and discovery using the results of previous steps. For preprocessing, this includes compensating for spectral overlap, transforming data onto scales conducive to visualization and analysis, assessing data for quality, and normalizing data across samples and experiments. For population identification, tools are available to aid traditional manual identification of populations in two-dimensional scatter plots (gating), to use dimensionality reduction to aid gating, and to find populations automatically in higher dimensional space in a variety of ways. It is also possible to characterize data in more comprehensive ways, such as the density-guided binary space partitioning technique known as probability binning, or by combinatorial gating. Finally, diagnosis using flow cytometry data can be aided by supervised learning techniques, and discovery of new cell types of biological importance by high-throughput statistical methods, as part of pipelines incorporating all of the aforementioned methods.

Open standards, data, and software are also key parts of flow cytometry bioinformatics. Data standards include the widely adopted Flow Cytometry Standard (FCS) defining how data from cytometers should be stored, but also several new standards under development by the International Society for Advancement of Cytometry (ISAC) to aid in storing more detailed information about experimental design and analytical steps. Open data is slowly growing with the opening of the CytoBank database in 2010 and FlowRepository in 2012, both of which allow users to freely distribute their data, and the latter of which has been recommended as the preferred repository for MIFlowCyt-compliant data by ISAC. Open software is most widely available in the form of a suite of Bioconductor packages, but is also available for web execution on the GenePattern platform.

This is a “Topic Page” article for *PLOS Computational Biology*.

## Flow Cytometry Data

**Figure 1:**
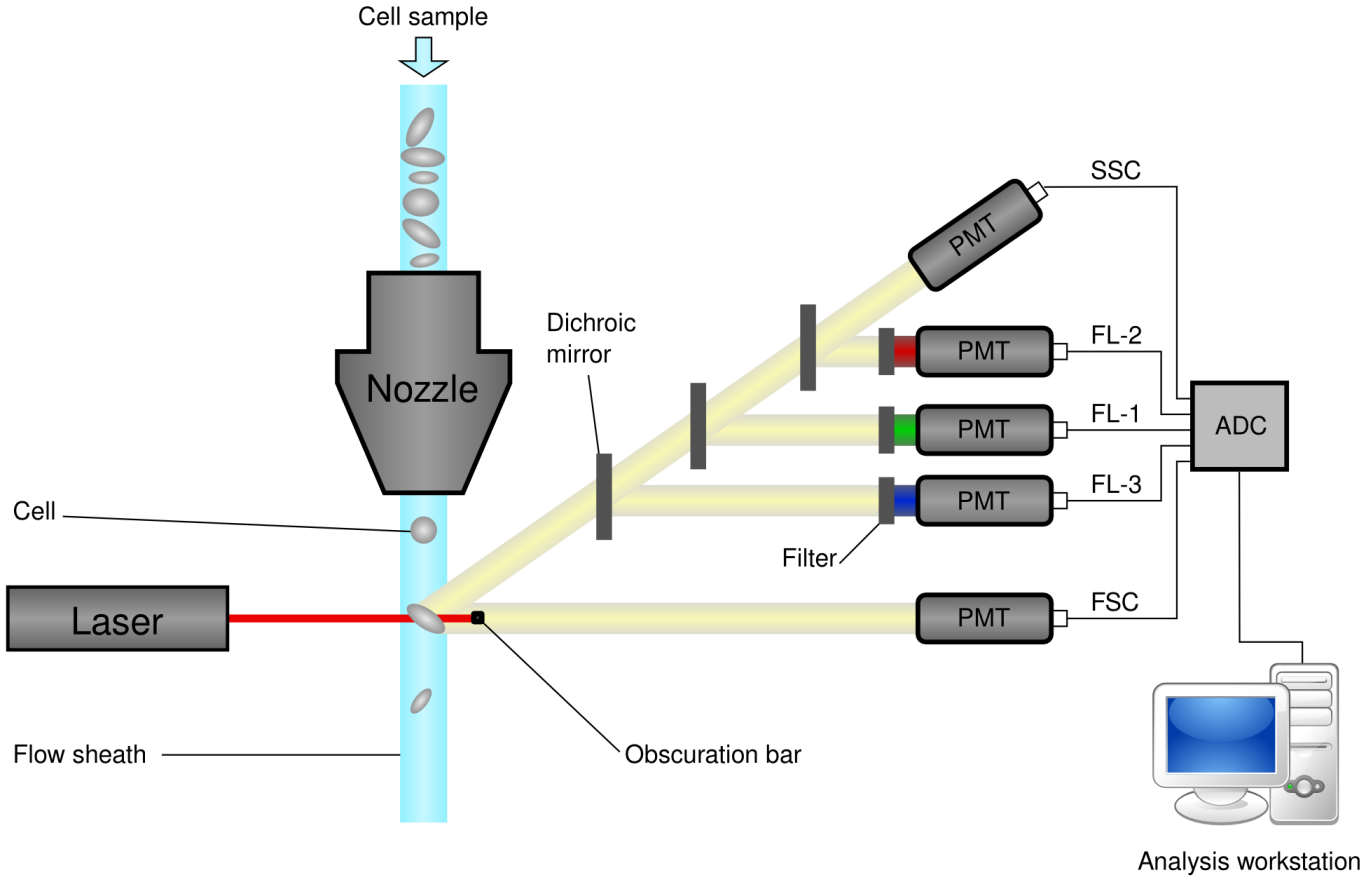
Schematic diagram of a flow cytometer, showing focusing of the fluid sheath, laser, optics (in simplified form, omitting focusing), photomultiplier tubes (PMTs), analogue-to-digital converter, and analysis workstation.

Flow cytometers operate by hydrodynamically focusing suspended cells so that they separate from each other within a fluid stream. The stream is passed by one or more lasers, and the resulting fluorescent and scattered light is detected by photomultipliers. By using optical filters, particular fluorophores on or within the cells can be quantified by peaks in their emission spectra. This process is illustrated in Figure 1. Reporter molecules may be endogenous fluorophores such as chlorophyll or transgenic green fluorescent protein, or they may be artificial fluorophores covalently bonded to detection molecules such as antibodies for detecting proteins, or hybridization probes for detecting DNA or RNA.

The ability to quantify these has led to flow cytometry being used in a wide range of applications, including but not limited to:

- Monitoring of CD4 count in HIV [1]
- Diagnosis of various cancers [2], [3]
- Analysis of aquatic microbiomes [4]
- Sperm sorting [5]
- Measuring telomere length [6]

Until the early 2000s, flow cytometry could only measure a few fluorescent markers at a time. Through the late 1990s into the mid-2000s, however, rapid development of new fluorophores resulted in modern instruments capable of quantifying up to 18 markers per cell [7]. More recently, the new technology of mass cytometry replaces fluorophores with rare earth elements detected by time of flight mass spectrometry, achieving the ability to measure the expression of 34 or more markers [8]. At the same time, microfluidic qPCR methods are providing a flow cytometry–like method of quantifying 48 or more RNA molecules per cell [9]. The rapid increase in the dimensionality of flow cytometry data coupled with the development of high-throughput robotic platforms capable of assaying hundreds to thousands of samples automatically have created a need for improved computational analysis methods [7].

## Steps in Computational Flow Cytometry Data Analysis

**Figure 2:**
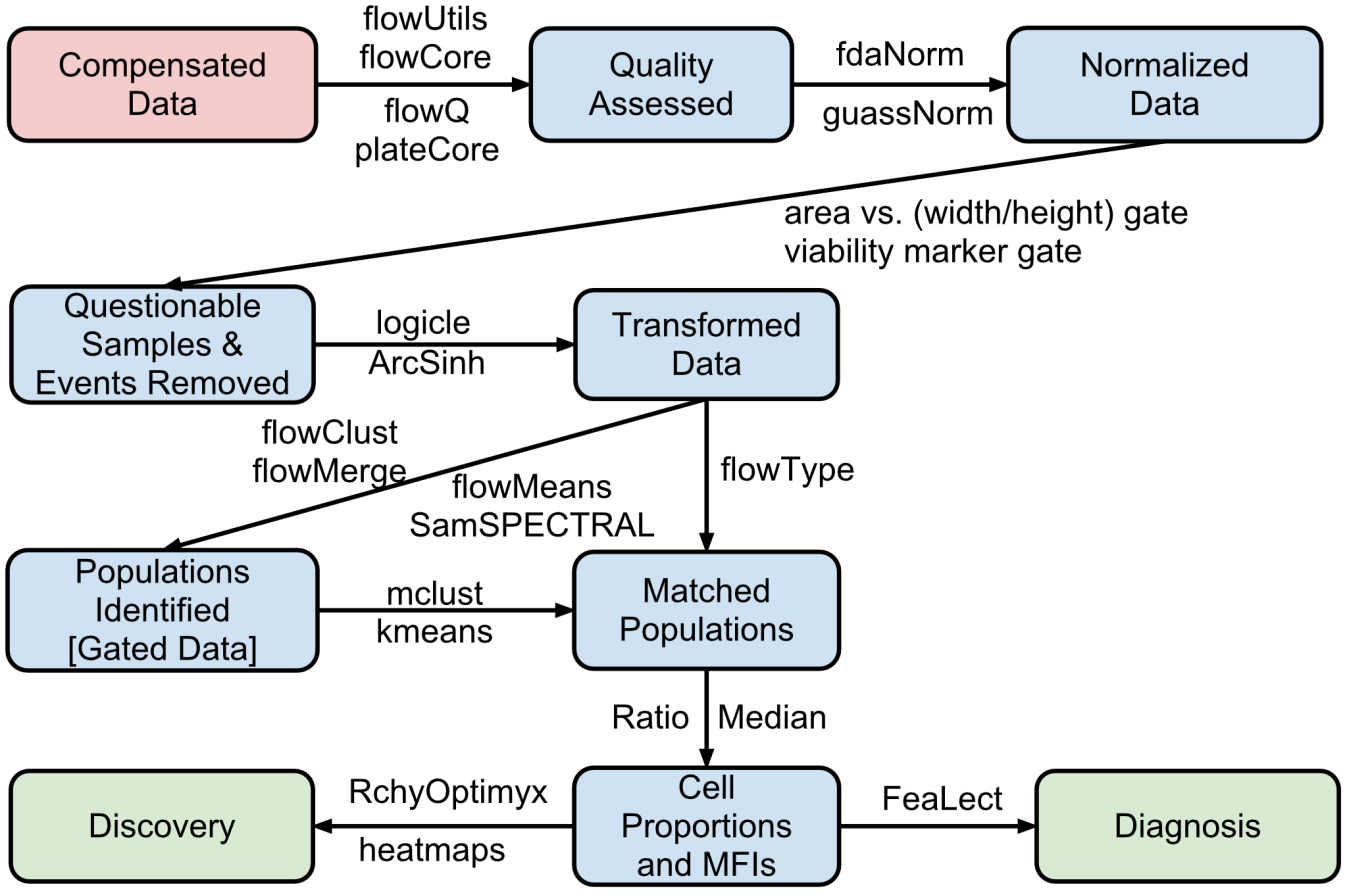
An example pipeline for analysis of FCM data and some of the Bioconductor packages relevant to each step.

The process of moving from primary FCM data to disease diagnosis and biomarker discovery (illustrated in Figure 2) involves four major steps:

1. Data preprocessing (including compensation, transformation, and normalization)
2. Cell population identification (a.k.a. gating)
3. Cell population matching for cross-sample comparison
4. Relating cell populations to external variables (diagnosis and discovery)

Saving of the steps taken in a particular flow cytometry workflow is supported by some flow cytometry software, and is important for the reproducibility of flow cytometry experiments. However, saved workspace files are rarely interchangeable between software [10]. An attempt to solve this problem is the development of the Gating-ML XML-based data standard (discussed in more detail in the data formats and interchange section), which is slowly being adopted in both commercial and open-source flow cytometry software [11].

## Data Preprocessing

### Compensation

When more than one fluorochrome is used with the same laser, their emission spectra frequently overlap. Each particular fluorochrome is typically measured using a bandpass optical filter set to a narrow band at or near the fluorochrome's emission intensity peak. The result is that the reading for any given fluorochrome is actually the sum of that fluorochrome's peak emission intensity, and the intensity of all other fluorochromes' spectra where they overlap with that frequency band. This overlap is termed spillover, and the process of removing spillover from flow cytometry data is called compensation [12].

Compensation is typically accomplished by running a series of representative samples each stained for only one fluorochrome, to give measurements of the contribution of each fluorochrome to each channel [12]. The total signal to remove from each channel can be computed by solving a system of linear equations based on this data to produce a spillover matrix, which when inverted and multiplied with the raw data from the cytometer produces the compensated data [12], [13]. The processes of computing the spillover matrix, or applying a precomputed spillover matrix to compensate flow cytometry data, are standard features of flow cytometry software [14].

### Transformation

Cell populations detected by flow cytometry are often described as having approximately log-normal expression [15]. As such, they have traditionally been transformed to a logarithmic scale. In early cytometers, this was often accomplished even before data acquisition by use of a log amplifier. On modern instruments, data is usually stored in linear form, and transformed digitally prior to analysis.

However, compensated flow cytometry data frequently contains negative values due to compensation, and cell populations do occur that have low means and normal distributions [16]. Logarithmic transformations cannot properly handle negative values, and poorly display normally distributed cell types [16], [17]. Alternative transformations that address this issue include the log-linear hybrid transformations Logicle [16], [18] and Hyperlog [19], as well as the hyperbolic arcsine and the Box-Cox [20].

A comparison of commonly used transformations concluded that the biexponential and Box-Cox transformations, when optimally parameterized, provided the clearest visualization and least variance of cell populations across samples [17]. However, a later comparison of the flowTrans package used in that comparison indicated that it did not parameterize the Logicle transformation in a manner consistent with other implementations, potentially calling those results into question [21].

### Quality Control

Particularly in newer, high-throughput experiments, there is a need for visualization methods to help detect technical errors in individual samples. One approach is to visualize summary statistics, such as the empirical distribution functions of single dimensions of technical or biological replicates to ensure they are the similar [22]. For more rigor, the KolmogorovSmirnov test can be used to determine if individual samples deviate from the norm [22]. The Grubbs test for outliers may be used to detect samples deviating from the group.

A method for quality control in higher-dimensional space is to use probability binning with bins fit to the whole dataset pooled together [23]. Then the standard deviation of the number of cells falling in the bins within each sample can be taken as a measure of multidimensional similarity, with samples that are closer to the norm having a smaller standard deviation [23]. With this method, higher standard deviation can indicate outliers, although this is a relative measure as the absolute value depends partly on the number of bins.

With all of these methods, the cross-sample variation is being measured. However, this is the combination of technical variations introduced by the instruments and handling, and actual biological information that is desired to be measured. Disambiguating the technical and the biological contributions to between-sample variation can be a difficult to impossible task [24].

### Normalization

Particularly in multicenter studies, technical variation can make biologically equivalent populations of cells difficult to match across samples. Normalization methods to remove technical variance, frequently derived from image registration techniques, are thus a critical step in many flow cytometry analyses. Single-marker normalization can be performed using landmark registration, in which peaks in a kernel density estimate of each sample are identified and aligned across samples [24].

## Identifying Cell Populations

A critical step in analysis of flow cytometric data is the identification of multidimensional regions that contain functionally and phenotypically homogeneous groups of cells for further analysis [27].

### Gating

The data generated by flow cytometers can be plotted in one or two dimensions to produce a histogram or scatter plot. The regions on these plots can be sequentially separated, based on fluorescence intensity, by creating a series of subset extractions, termed “gates.” These gates can be produced using software, e.g., FlowJo [28], FCS Express [29], WinMDI [30], CytoPaint (aka Paint-A-Gate) [31], VenturiOne, CellQuest Pro, Cytospec [32], or Kaluza [33].

**Figure 3:**
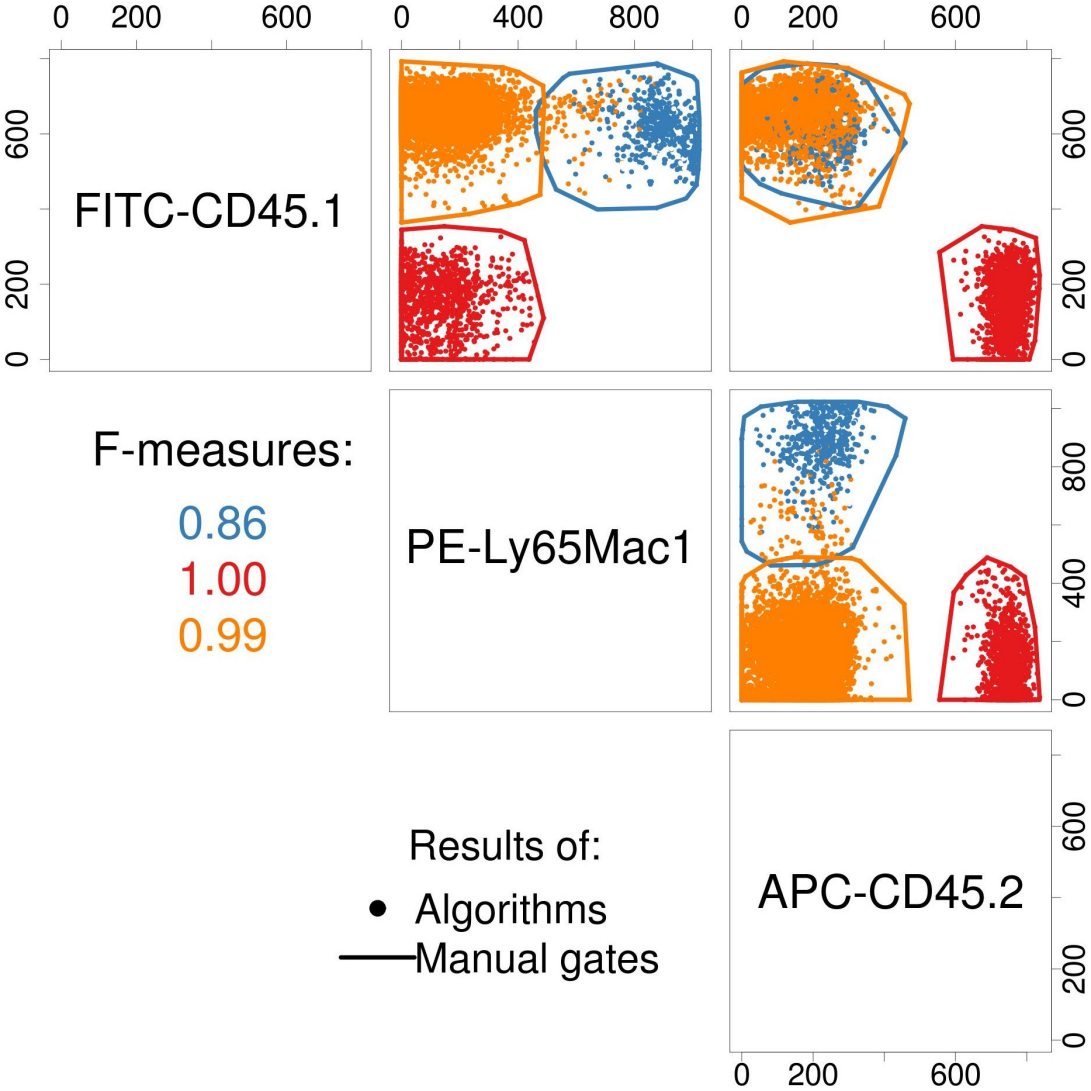
Comparison of consensus of eight independent manual gates (polygons) and automated gates (colored dots). The consensus of the manual gates and the algorithms were produced using the CLUE package [25]. Figure reproduced with permission from [26].

In datasets with a low number of dimensions and limited cross-sample technical and biological variability (e.g., clinical laboratories), manual analysis of specific cell populations can produce effective and reproducible results. However, exploratory analysis of a large number of cell populations in a high-dimensional dataset is not feasible [34]. In addition, manual analysis in less controlled settings (e.g., cross-laboratory studies) can increase the overall error rate of the study [35]. In one study, several computational gating algorithms performed better than manual analysis in the presence of some variation [26] (illustrated in Figure 3). However, despite the considerable advances in computational analysis, manual gating remains the main solution for the identification of specific rare cell populations that are not well-separated from other cell types.

#### Gating guided by dimension reduction

**Figure 4:**
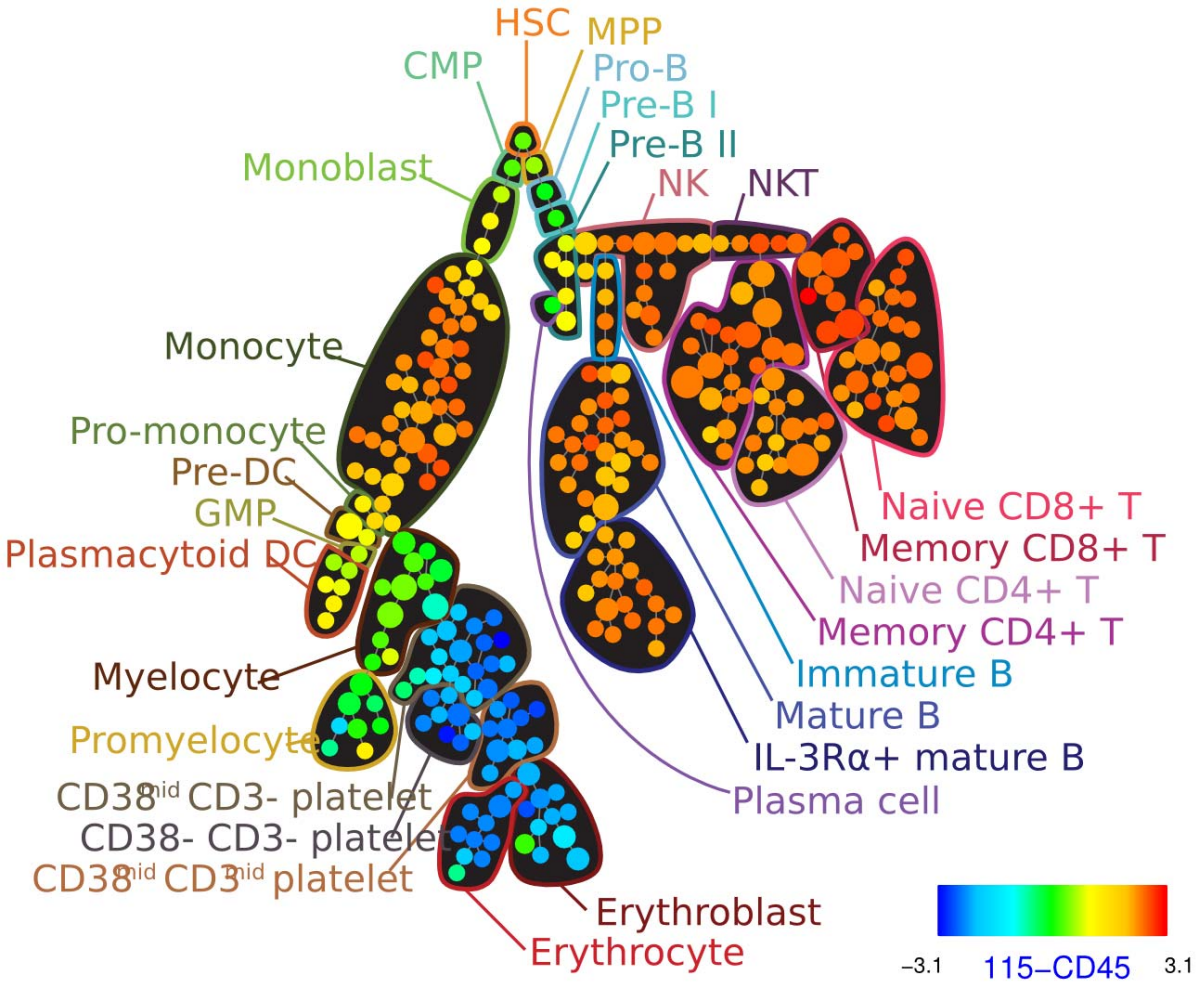
Cell populations in a high-dimensional mass-cytometry dataset manually gated after dimension reduction using 2-D layout for a minimum spanning tree. Figure reproduced from the data provided in [40].

As the number of markers measured by flow cytometry increases, the number of scatter plots that need to be investigated increases exponentially (some markers need to be investigated several times for each group of cells to resolve high-dimensional differences between cell types that appear to be similar in most markers) [36]. To address this issue, principal component analysis has been used to summarize the high-dimensional datasets using a combination of markers that maximizes the variance of all data points [37]. However, PCA is a linear method and is not able to preserve complex and non-linear relationships. More recently, two-dimensional minimum spanning tree layouts have been used to guide the manual gating process (illustrated in Figure 4). Density-based down-sampling and clustering was used to better represent rare populations and control the time and memory complexity of the minimum spanning tree construction process [38]. More sophisticated dimension reduction algorithms are yet to be investigated [39].

#### Automated gating

Developing computational tools for identification of cell populations has been an area of active research only since 2008. Many individual clustering approaches have recently been developed, including model-based algorithms (e.g., flowClust [41] and FLAME [42]), density-based algorithms (e.g., FLOCK [43] and SWIFT), graph-based approaches (e.g., SamSPECTRAL [44]), and, most recently, hybrids of several approaches (flowMeans [45] and flowPeaks [46]). These algorithms are different in terms of memory and time complexity, their software requirements, their ability to automatically determine the required number of cell populations, and their sensitivity and specificity. The FlowCAP (Flow Cytometry: Critical Assessment of Population Identification Methods) project, with active participation from most academic groups with research efforts in the area, is providing a way to objectively cross-compare state-of-the-art automated analysis approaches [26].

### Probability Binning Methods

**Figure 5:**
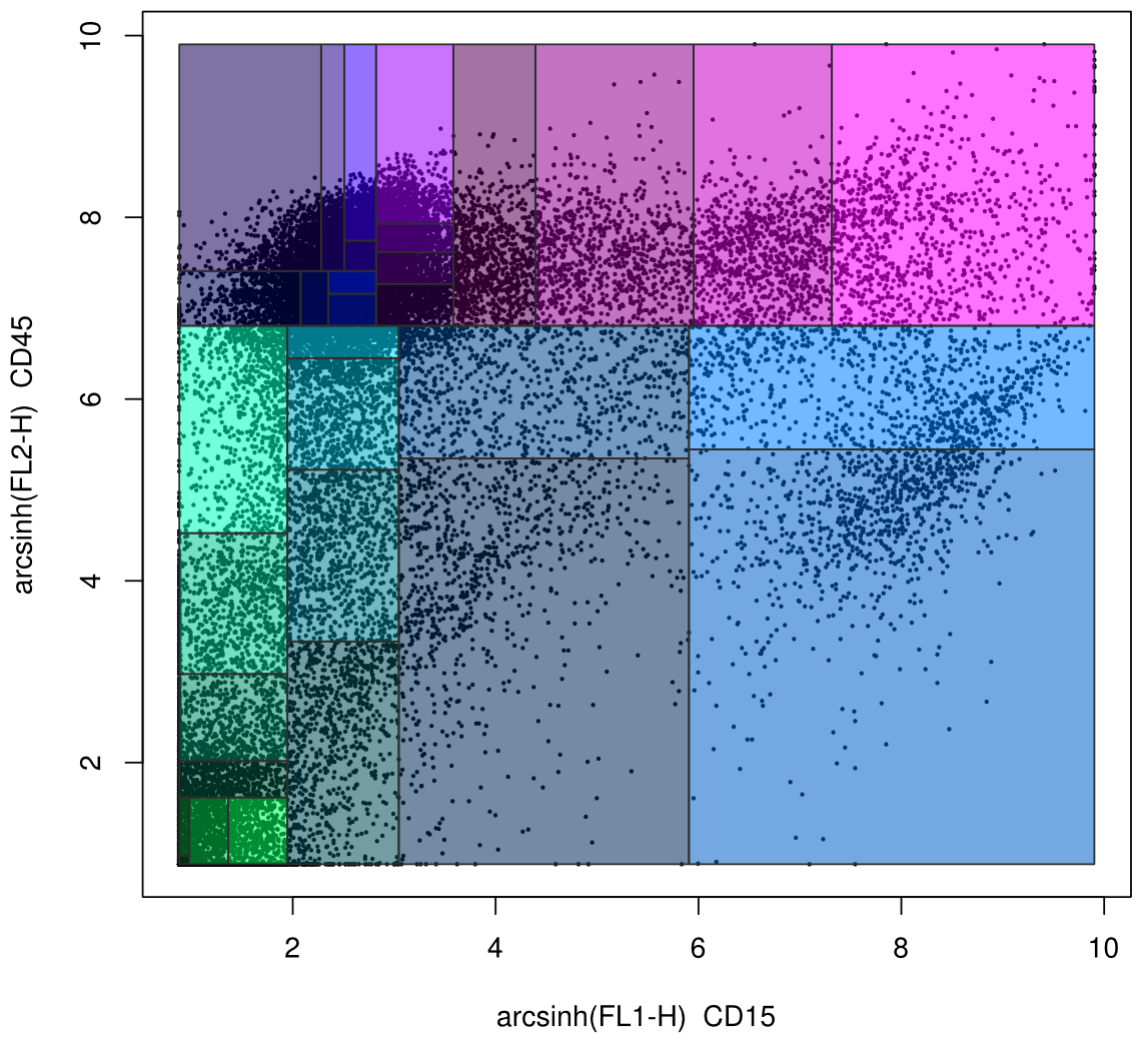
An example of probability binning, created using the flowFP Bioconductor package. The dots represent individual events in an FCS file. The rectangles represent the bins.

Probability binning is a non-gating analysis method in which flow cytometry data is split into quantiles on a univariate basis (shown in Figure 5) [47]. The locations of the quantiles can then be used to test for differences between samples using the chi-squared test [47].

This was later extended into multiple dimensions in the form of frequency difference gating, a binary space partitioning technique in which data is iteratively partitioned along the median [48]. These partitions (or bins) are fit to a control sample. Then the proportion of cells falling within each bin in test samples can be compared to the control sample by the chi-squared test.

Finally, cytometric fingerprinting uses a variant of frequency difference gating to set bins and measure for a series of samples how many cells fall within each bin [23]. These bins can be used as gates and used for subsequent analysis similarly to automated gating methods.

### Combinatorial Gating

High-dimensional clustering algorithms are often unable to identify rare cell types that are not well separated from other major populations. Matching these small cell populations across multiple samples is even more challenging. In manual analysis, prior biological knowledge (e.g., biological controls) provides guidance to reasonably identify these populations. However, integrating this information into the exploratory clustering process (e.g., as in semi-supervised learning) has not been successful.

An alternative to high-dimensional clustering is to identify cell populations using one marker at a time and then combine them to produce higher-dimensional clusters. This functionality was first implemented in FlowJo [28]. The flowType algorithm builds on this framework by allowing the exclusion of the markers [49]. This enables the development of statistical tools (e.g., RchyOptimyx) that can investigate the importance of each marker and exclude high-dimensional redundancies [50].

## Diagnosis and Discovery

After identification of the cell population of interest, a cross-sample analysis can be performed to identify phenotypical or functional variations that are correlated with an external variable (e.g., a clinical outcome). These studies can be partitioned into two main groups:

### Diagnosis

In these studies, the goal usually is to diagnose a disease (or a sub-class of a disease) using variations in one or more cell populations. For example, one can use multidimensional clustering to identify a set of clusters, match them across all samples, and then use supervised learning to construct a classifier for prediction of the classes of interest (e.g., this approach can be used to improve the accuracy of the classification of specific lymphoma subtypes [51]). Alternatively, all the cells from the entire cohort can be pooled into a single multidimensional space for clustering before classification [52]. This approach is particularly suitable for datasets with a high amount of biological variation (in which cross-sample matching is challenging) but requires technical variations to be carefully controlled [53].

### Discovery

**Figure 6:**
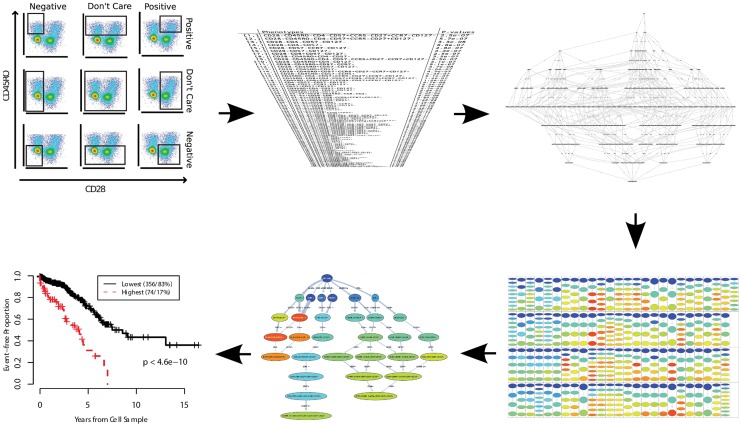
Overview of the flowType/RchyOptimyx pipeline for identification of correlates of protection against HIV. First, tens of thousands of cell populations are identified by combining one-dimensional partitions (panel 1). The cell populations are then analyzed using a statistical test (and Bonferroni's method for multiple testing correction) to identify those correlated with the survival information. Panel 3 shows a complete gating hierarchy describing all possible strategies for gating that cell population. This graph can be mined to identify the “best” gating strategy (i.e., the one in which the most important markers appear earlier). These hierarchies for all selected phenotypes are demonstrated in panel 4. In panel 5, these hierarchies are merged into a single graph that summarizes the entire dataset and demonstrates the trade-off between the number of markers involved in each phenotype and the significance of the correlation with the clinical outcome (e.g., as measured by the KaplanMeier estimator in panel 6). Figure reproduced in part from [49] (public domain) and [50].

In a discovery setting, the goal is to identify and describe cell populations correlated with an external variable (as opposed to the diagnosis setting in which the goal is to combine the predictive power of multiple cell types to maximize the accuracy of the results). Similar to the diagnosis use case, cluster matching in high-dimensional space can be used for exploratory analysis, but the descriptive power of this approach is very limited, as it is hard to characterize and visualize a cell population in a high-dimensional space without first reducing the dimensionality [52], [54]. Finally, combinatorial gating approaches have been particularly successful in exploratory analysis of FCM data. Simplified Presentation of Incredibly Complex Evaluations (SPICE) is a software package that can use the gating functionality of FlowJo to statistically evaluate a wide range of different cell populations and visualize those that are correlated with the external outcome. flowType and RchyOptimyx (as previously discussed) expand this technique by adding the ability of exploring the impact of independent markers on the overall correlation with the external outcome. This enables the removal of unnecessary markers and provides a simple visualization of all identified cell types. In a recent analysis of a large (n = 466) cohort of HIV+ patients, this pipeline identified three correlates of protection against HIV, only one of which had been previously identified through extensive manual analysis of the same dataset (as illustrated in Figure 6) [49].

## Data Formats and Interchange

### Flow Cytometry Standard

**Figure 7:**
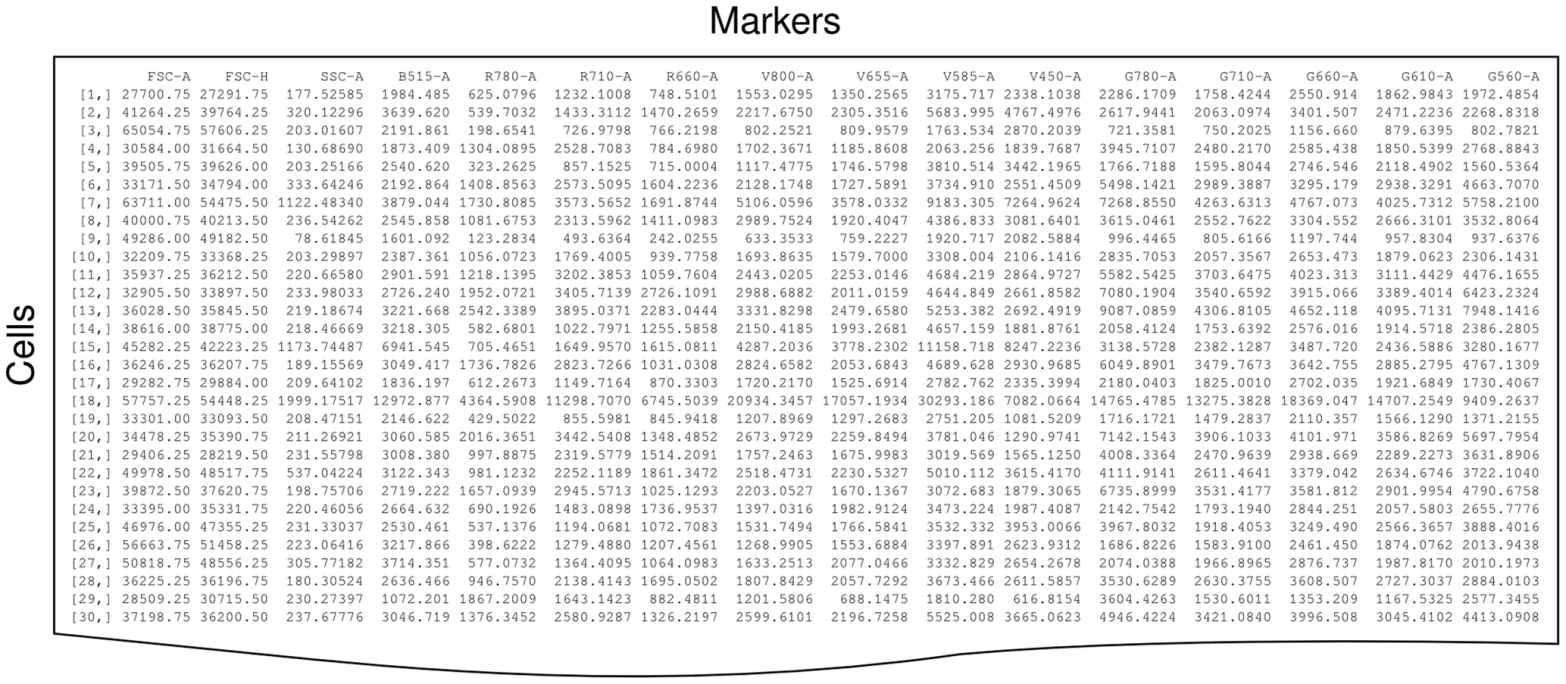
Representation of flow cytometry data from an instrument with three scatter channels and 13 fluorescent channels. Only the values for the first 30 (of hundreds of thousands) of cells are shown.

Flow cytometry data is typically saved for analysis in the form of an array, with fluorescence and scatter channels represented in columns and individual “events” (most of which are cells) forming the rows, as shown in Figure 7. The number of events acquired from each sample usually ranges between the low thousands and the low millions.

The first version of a Flow Cytometry Standard (FCS) was developed in 1984 [55]. Since then, FCS has become the standard file format supported by all flow cytometry software and hardware vendors. FCS is a binary file format with three main segments: a text segment containing metadata in keyword/value pairs structures, a data segment usually containing a matrix of detected expression values (so-called list mode format), and a rarely used analysis segment. The FCS specification has traditionally been developed and maintained by the International Society for Advancement of Cytometry (ISAC) [56].

Over the years, updates have been incorporated to adapt to technological advancements in both flow cytometry and computing technologies.

In 1990, FCS 2.0 was introduced [57]. Features introduced in FCS 2.0 included the option of multiple datasets within a data file, the use of different byte orders accommodating hardware variations on different computing platforms, and basic compensation and scaling information. FCS 2.0 was followed by FCS 3.0 in 1997, which introduced the possibility of storing datasets larger than 100 MB [58].

The latest version, FCS 3.1, was introduced in 2010 [59]. It retains the basic FCS file structure and most features of previous versions of the standard. Changes included in FCS 3.1 address potential ambiguities in the previous versions and provide a more robust standard. They include simplified support for international characters and improved support for storing compensation. The major additions are support for preferred display scale, a standardized way of capturing the sample volume, information about the origins of the data file, and support for plate and well identification in high-throughput, plate-based experiments.

FCS used to be the only widely adopted file format in flow cytometry. Recently, additional standard file formats have been developed by ISAC.

### netCDF

ISAC is considering replacing FCS with a flow cytometry–specific version of the Network Common Data Form (netCDF) file format [60]. netCDF is a set of freely available software libraries and machine-independent data formats that support the creation, access, and sharing of array-oriented scientific data. In 2008, ISAC drafted the first version of netCDF conventions for storage of raw flow cytometry data [61].

### Archival Cytometry Standard (ACS)

The Archival Cytometry Standard (ACS) is being developed to bundle data with different components describing cytometry experiments [62]. It captures relations among data, metadata, analysis files, and other components, and includes support for audit trails, versioning, and digital signatures. The ACS container is based on the ZIP file format with an XML-based table of contents specifying relations among files in the container. The XML Signature W3C Recommendation has been adopted to allow for digital signatures of components within the ACS container. An initial draft of ACS was designed in 2007 and finalized in 2010. Since then, ACS support has been introduced in several software tools including FlowJo and Cytobank.

### Gating-ML

The lack of gating interoperability has traditionally been a bottleneck preventing reproducibility of flow cytometry data analysis and the usage of multiple analytical tools. To address this shortcoming, ISAC developed Gating-ML, an XML-based mechanism to formally describe gates and related data (scale) transformations [10]. The draft recommendation version of Gating-ML was approved by ISAC in 2008, and it is partially supported by tools like FlowJo, the flowUtils library in R/Bioconductor, and FlowRepository [62]. It supports rectangular gates, polygon gates, convex polytopes, ellipsoids, decision trees, and Boolean collections of any of the other types of gates. In addition, it includes dozens of built-in public transformations that have been shown to be potentially useful for display or analysis of cytometry data. In 2013, Gating-ML version 2.0 was approved by ISAC's Data Standards Task Force as a Recommendation. This new version offers slightly less flexibility in terms of the power of gating description; however, it is also significantly easier to implement in software tools [11].

### Classification Results (CLR)

The Classification Results (CLR) File Format [63] has been developed to exchange the results of manual gating and algorithmic classification approaches in a standard way in order to be able to report and process the classification. CLR is based on the commonly supported CSV file format with columns corresponding to different classes and cell values containing the probability of an event being a member of a particular class. These are captured as values between 0 and 1. Simplicity of the format and its compatibility with common spreadsheet tools have been the major requirements driving the design of the specification. Although it was originally designed for the field of flow cytometry, it is applicable in any domain that needs to capture either fuzzy or unambiguous classifications of virtually any kinds of objects.

## Public Data and Software

As in other bioinformatics fields, development of new methods has primarily taken the form of free open-source software, and several databases have been created for depositing open data.

### Bioconductor

The Bioconductor project is a repository of free open-source software, mostly written in the R programming language [64]. As of July 2013, Bioconductor contained 21 software packages for processing flow cytometry data [65]. These packages cover most of the range of functionality described earlier in this article.

### GenePattern

GenePattern is a predominantly genomic analysis platform with over 200 tools for analysis of gene expression, proteomics, and other data. A web-based interface provides easy access to these tools and allows the creation of automated analysis pipelines enabling reproducible research. Recently, a GenePattern Flow Cytometry Suite has been developed in order to bring advanced flow cytometry data analysis tools to experimentalists without programmatic skills. It contains close to 40 open-source GenePattern flow cytometry modules covering methods from basic processing of flow cytometry standard (i.e., FCS) files to advanced algorithms for automated identification of cell populations, normalization, and quality assessment. Internally, most of these modules leverage from functionality developed in Bioconductor.

Much of the functionality of the Bioconductor packages for flow cytometry analysis has been packaged up for use with the GenePattern [66] workflow system, in the form of the GenePattern Flow Cytometry Suite [67].

### Public Databases

The Minimum Information about a Flow Cytometry Experiment (MIFlowCyt) requires that any flow cytometry data used in a publication be available, although this does not include a requirement that it be deposited in a public database [68]. Thus, although the journals Cytometry A and B, as well as all journals from the Nature Publishing Group require MIFlowCyt compliance, there is still relatively little publicly available flow cytometry data. Some efforts have been made toward creating public databases, however.

Firstly, CytoBank, which is a complete web-based flow cytometry data storage and analysis platform, has been made available to the public in a limited form [69]. Using the CytoBank code base, FlowRepository was developed in 2012 with the support of ISAC to be a public repository of flow cytometry data [70]. FlowRepository facilitates MIFlowCyt compliance [71], and as of July 2013 contained 65 public datasets [72].

### Datasets

In 2012, the flow cytometry community started to release a set of publicly available datasets. A subset of these datasets representing the existing data analysis challenges is described below. For comparison against manual gating, the FlowCAP-I project has released five datasets, manually gated by human analysts, and two of them gated by eight independent analysts [26]. The FlowCAP-II project included three datasets for binary classification and also reported several algorithms that were able to classify these samples perfectly. FlowCAP-III included two larger datasets for comparison against manual gates as well as one more challenging sample classification dataset. As of March 2013, public release of FlowCAP-III was still in progress [73]. The datasets used in FlowCAP-I, II, and III either have a low number of subjects or parameters. However, recently several more complex clinical datasets have been released including a dataset of 466 HIV-infected subjects, which provides both 14 parameter assays and sufficient clinical information for survival analysis [50], [74]–[76].

Another class of datasets are higher-dimensional mass cytometry assays. A representative of this class of datasets is a study that includes analysis of two bone marrow samples using more than 30 surface or intracellular markers under a wide range of different stimulations [8]. The raw data for this dataset is publicly available as described in the manuscript, and manual analyses of the surface markers are available upon request from the authors.

## Open Problems

Despite rapid development in the field of flow cytometry bioinformatics, several problems remain to be addressed.

Variability across flow cytometry experiments arises from biological variation among samples, technical variations across instruments used, as well as methods of analysis. In 2010, a group of researchers from Stanford University and the National Institutes of Health pointed out that while technical variation can be ameliorated by standardizing sample handling, instrument setup, and choice of reagents, solving variation in analysis methods will require similar standardization and computational automation of gating methods [77]. They further opined that centralization of both data and analysis could aid in decreasing variability between experiments and in comparing results [77].

This was echoed by another group of Pacific Biosciences and Stanford University researchers, who suggested that cloud computing could enable centralized, standardized, high-throughput analysis of flow cytometry experiments [78]. They also emphasized that ongoing development and adoption of standard data formats could continue to aid in reducing variability across experiments [78]. They also proposed that new methods will be needed to model and summarize results of high-throughput analysis in ways that can be interpreted by biologists [78], as well as ways of integrating large-scale flow cytometry data with other high-throughput biological information, such as gene expression, genetic variation, metabolite levels, and disease states [78].

## Supporting Information

Text S1 Version history of the text file.
(XML)

Text S2 Peer reviews and response to reviews. Human-readable versions of the reviews and authors' responses are available as comments on this article.
(XML)
